# Noninvasive monitoring and prediction of cell count of *Saccharomyces* suspensions using ultrasonic measurements and artificial neural networks

**DOI:** 10.1007/s00216-025-06175-6

**Published:** 2025-11-21

**Authors:** Dominik Geier, Michael Metzenmacher, Iain Whitehead, Thomas Becker

**Affiliations:** Chair of Brewing and Beverage Technology, TUM School of Life Sciences, Technical University of Munich, Weihenstephaner Steig 20, 85354 Freising, Germany

**Keywords:** Noninvasive monitoring, Cell count, *Saccharomyces*, Ultrasonic measurements, Artificial neural networks

## Abstract

The dynamic nature of biological suspensions during fermentation requires continuous monitoring. In *Saccharomyces* suspensions, cell count directly influences biomass production and is an indicator of metabolic efficiency. However, established methods to monitor cell count require sampling, are invasive, or are restricted by detection limits. In contrast, ultrasound techniques are noninvasive and provide real-time feedback. In this study, ultrasound was used to analyze yeast suspensions with increasing concentrations (0.0–1.0 wt%) from three *Saccharomyces* strains, each assessed at three wort concentrations (10, 12, and 14 wt% extract). The collected analytical data, consisting of manually determined cell count and nine ultrasound-derived features, were used to develop separate artificial neural network (ANN) models for cell count prediction. Three strain-specific regression models were initially developed; their evaluation metrics confirmed that the selected ANN architecture reliably predicted cell count for each strain (*R*^2^ = 0.96–0.98). Subsequently, regression models able to predict cell count across all strains were developed. The performance of these regression models progressively improved with the integration of non-ultrasound data into the input layer (*R*^2^ = 0.92–0.97). Additionally, a separate classification model was developed to distinguish between yeast strains, using solely the nine ultrasound-derived features. This classification model achieved an overall accuracy of 96.6%, demonstrating high generalization performance across different biological and process conditions. These findings confirmed that ultrasound-derived features could be effectively modeled using artificial neural networks for cell count prediction and characterization of *Saccharomyces* suspensions.

**Graphical Abstract:**

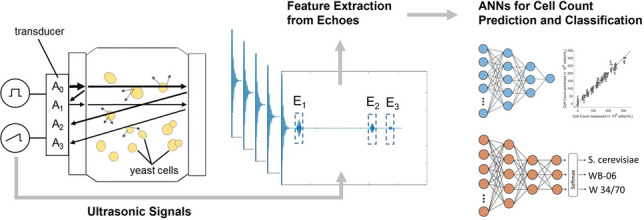

**Supplementary Information:**

The online version contains supplementary material available at 10.1007/s00216-025-06175-6.

## Introduction

Biomass concentration, particularly yeast cell count, is a critical control parameter in fermentation processes which directly influences biomass production, metabolic efficiency, and final product quality. Therefore, yeast cell count must be carefully controlled throughout all process phases, including propagation, inoculation, and fermentation. Alignment between metabolic demand and substrate availability is essential for maximizing process efficiency. Failure to maintain this balance may compromise process stability and result in excessively high or insufficiently low yeast concentrations. Excessive yeast concentrations may result in increased oxygen demand and accelerated nutrient depletion. In contrast, low cell concentrations are characterized by reduced metabolic activity and product yields. In addition, insufficient biomass can lead to prolonged fermentations and a higher risk of microbial contamination. Overall, maintaining optimal yeast cell counts promotes process efficiency, stable operation, and consistent reproducibility. However, in *Saccharomyces* systems, optimal cell counts vary depending on multiple factors, such as growth phase and specific process conditions [1, 2].

Although accurate and continuous cell count monitoring allows precise control over fermentation kinetics, product yield, and suppression of undesirable by-products, the existing methods suffer from inherent limitations. Due to their simplicity and low cost, turbidity measurements are widely used for estimating optical cell density. However, their accuracy is influenced by factors such as yeast cell size, morphology, and aggregation, as well as the optical properties of the suspension [3]. In densely concentrated or opaque suspensions, turbidity often lacks linearity; therefore, to remain within reliable measurement ranges, sample dilution is required [4, 5]. Furthermore, regular calibration is required [6], and long-term reliability is compromised by probe fouling [7]. In contrast, gravimetric methods provide direct biomass quantification but are time-consuming, require large sample volumes, and are limited at low concentrations [8]. Another common method is flow cytometry, which offers detailed yeast population analysis but is costly, labor-intensive, and demands sampling [9, 10]. Similarly, Coulter counters quantify cell concentration via impedance changes, but are affected by medium conductivity and sample preparation constraints, thus limiting their real-time application [11, 12]. Manual microscopy using hemocytometers remains the standard method for yeast cell count determination, but it is off-line, operator-dependent, and time-consuming [13]. All these different methods offer individual advantages; however, common limitations include being invasive, prone to contamination risks, time-consuming, and providing limited information on the dynamic properties of yeast suspensions. As a result, there is a growing interest in noninvasive, real-time techniques that can address and resolve these challenges. In-line alternatives for continuous monitoring of yeast cell concentrations include Raman spectroscopy and in situ microscopy (ISM). Raman spectroscopy has been shown to improve ethanol fermentation yields through optimized process control [14, 15]. However, weak Raman signals, interference from biomolecular fluorescence, and high instrumentation costs are some of its limitations [16–19]. Alternatively, ISM allows direct visualization of yeast concentration and morphology, but sensitivity declines above 4.5 × 10^6^ cells/mL and is further limited by low-contrast or small cells [20, 21].

Unlike Raman spectroscopy or ISM, ultrasonic waves effectively propagate through turbid media and interact with both soluble and insoluble substances. Recent studies have shown that ultrasound can determine sugar and ethanol concentrations in defined media. Schöck and Becker [22] quantified sucrose and ethanol by measuring sound velocity at two different temperatures using an ultrasonic sensor array, while Resa et al. [23], Vatandas et al. [24], and Hoche et al. [25] published similar findings for sugar–alcohol mixtures. In more complex systems, such as cell suspensions, measurable acoustic properties include attenuation (i.e., signal amplitude loss) and sound velocity, as these reflect changes in cell density and composition [26, 27]. Several studies have used ultrasonic techniques under controlled laboratory conditions to characterize dispersed cells in different media. High-frequency backscattering was used by Chen et al. [28] and Elvira et al. [29] to quantify red blood cells and yeast cells, respectively. Rodriguez-Molares et al. [30] proposed an empirical model for cyanobacteria concentration, but their invasive setup limits bioprocess applicability. More recently, Akbari et al. [31] developed a noninvasive Doppler sensor for real-time monitoring of Chinese hamster ovary cell concentration. Different *Saccharomyces* strains, suspended in Ringer solution, were examined by Geier et al. [32] using an ultrasonic pulse-echo measurement setup. Their data revealed an increase in attenuation and sound velocity with higher yeast concentrations.

Ultrasound is a noninvasive technology that can detect signal variations resulting from changes in biomass concentration (e.g., yeast cell count) or shifts in medium composition (e.g., wort concentration); however, data management and processing can be demanding. Given the complexity of ultrasound data and their interpretation, particularly in dense, heterogeneous yeast suspensions, machine learning (ML) techniques are increasingly being explored [33]. Among the available ML approaches, artificial neural networks (ANNs) do not rely on predefined assumptions and can model complex nonlinear patterns in acoustic signaling. ANNs have been successfully integrated into various stages of bioprocessing, including monitoring, control, and process optimization, thereby demonstrating their broad applicability and effectiveness in improving process outcomes [34–36]. Therefore, ANNs are well suited for ultrasound-based monitoring of bioprocesses, where ultrasonic wave propagation is highly application-dependent [37]. ANNs trained on labeled datasets relating ultrasound-derived features to known biomass concentrations can learn these relationships and generalize to accurately predict strain-specific properties in new, unseen data.

Before developing data-driven ML prediction models, it is necessary to generate a representative dataset that includes variation in both biological and process properties, while also exploring their interactive effects on ultrasonic wave propagation. In this study, three *Saccharomyces* strains commonly used in industrial applications were selected, and ultrasound measurements were performed on yeast suspensions with increasing concentrations (0.0–1.0 weight percent; wt%). Moreover, to represent variability in fermentation process conditions, concentration gradients for each yeast strain were also prepared in three separate worts with extract concentrations of 10, 12, and 14 wt%. For all samples, yeast cell counts were manually determined, and the recorded ultrasound signals were processed to extract relevant features. These ultrasound-derived features served as input features for training and evaluating the ANN regression models used for yeast cell count prediction. To improve the predictive performance of the models, a stepwise integration of additional encoded input features was evaluated. In this process, non-ultrasound variables of increasing specificity were progressively introduced, beginning with nominal strain identity and followed by ordinal wort concentration. In addition to the regression models for cell count prediction, a separate ANN model was developed for yeast strain classification. This classification model used only the nine ultrasound-derived features and expanded the output layer to allow multi-class classification of the different yeast strains. This methodology allowed a comprehensive assessment of the integration of noninvasive ultrasonic measurement techniques with machine learning algorithms for predicting cell count and yeast properties in complex fermentation media.

## Materials and methods

### Design and working principle of the experimental ultrasound setup

**Fig. 1:**
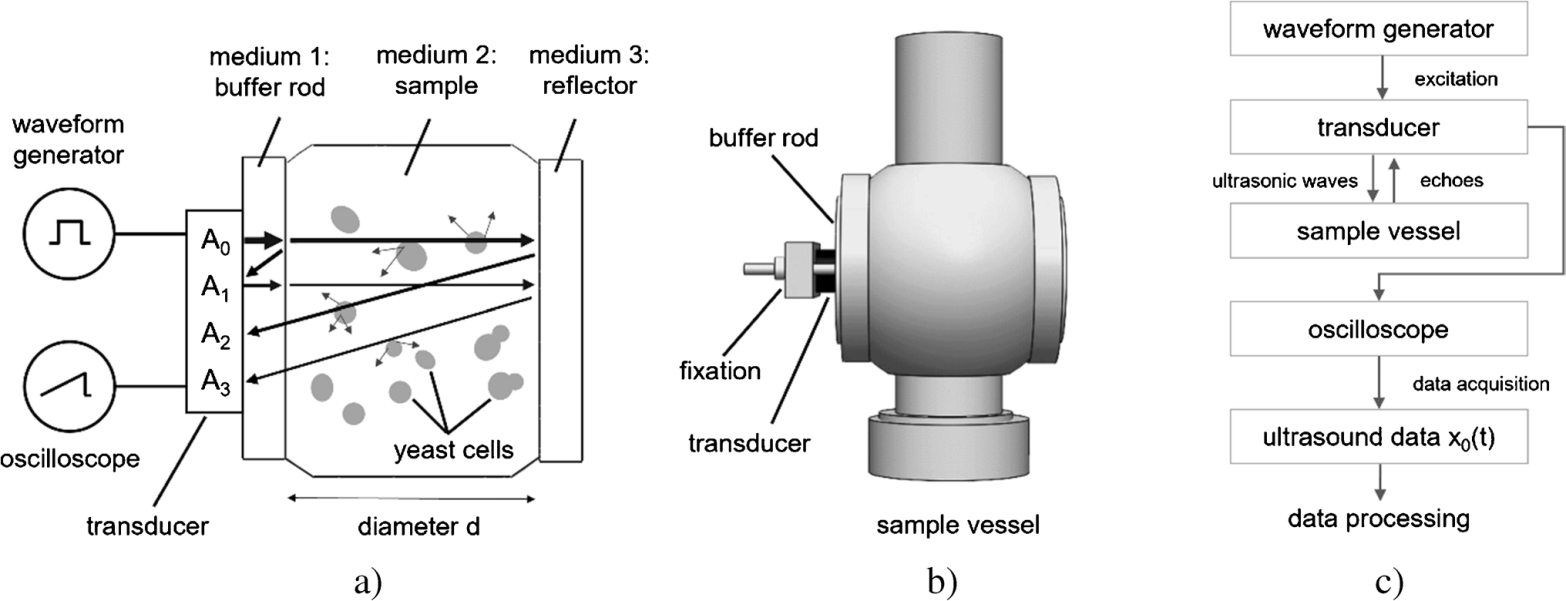
Schematic illustration of ultrasonic wave propagation in the experimental ultrasound setup (**a**), technical drawing of the sample vessel (**b**), and flowchart illustrating the ultrasound signal path from excitation to recording of the raw ultrasound data, ${x}_{0}(t)$ (**c**). In **a**, arrows indicate the path of the ultrasonic wave propagation and its interactions with different media. The waveform generator excites the transducer, thereby generating the initial ultrasonic wave with amplitude A_0_. This wave propagates through the buffer rod into the sample, where multiple reflections and interactions occur. The first echo, E_1_, arises at the buffer rod–sample interface; the second echo, E_2_, is generated at the sample–reflector interface; and a third echo, E_3_, results from the wave traversing the sample a second time. All echoes are captured by the transducer and recorded using an oscilloscope, yielding amplitudes A_1_, A_2_, and A_3_

The experimental ultrasound setup consisted of the electronic components used for both ultrasonic signal excitation and acquisition, as well as the sample vessel (see Fig. 1). A Varivent^®^ process connector was used as the sample vessel, suitable for hygienic applications and allowing integration of measurement and control instruments. The stainless-steel sample vessel included two Plexiglas^®^ plates that enclosed the buffer rod (i.e., medium 1) and the parallel reflector (i.e., medium 3); see Fig. 1a. The buffer rod had a diameter, *d*_b_, of 17 mm, and the sample vessel had a diameter, *d*, of 37.5 mm. To ensure a leak-tight closure, the bottom of the Varivent^®^ process connection was secured with a blind nut. The waveform generator (Agilent 35522 A, Keysight Technologies, Santa Rosa, CA, USA) produced a 10 Vpp pulse with a four-cycle square wave excitation. This electrical pulse was directly applied to a 2 MHz piezoelectric ultrasonic transducer (MB 2 SE, GE, Boston, MA, USA), which was mechanically coupled to the buffer rod (see Fig. 1b). The transducer functioned as both transmitter and receiver of ultrasonic signals, generating and detecting ultrasonic waves within the experimental setup. The buffer rod served as an acoustic delay line, which enabled transmitting the produced ultrasonic wave into the sample medium (i.e., medium 2) and temporal separation of the echoes. For effective ultrasound transmission into the sample, coupling gel (Aquasonic^®^ 100, Parker Laboratories Inc., Fairfield, NJ, USA) was used. The reflected echoes were captured with the same transducer and recorded directly by an oscilloscope (PicoScope 5204, Pico Technology, Cambridgeshire, UK) without any additional analog amplification or filtering. Data acquisition was performed using PicoScope 6 software (Pico Technology, Cambridgeshire, UK) at a sampling rate of 1 GHz. The schematic configuration and signal path of the experimental ultrasound setup are summarized in Fig. 1c.

The cross-sectional schematic of the sample vessel in Fig. 1a illustrates the propagation of the ultrasonic wave through the three sequentially layered media: the buffer rod (medium 1), the yeast suspension sample (medium 2), and the reflector (medium 3). The ultrasonic signal is generated by a 2 MHz transducer, which is excited by the waveform generator. The initial ultrasonic wave, with amplitude A_0_, propagates through the buffer rod and reaches the buffer rod–sample interface. At this interface, a portion of the wave is reflected, forming the first echo, E_1_, with amplitude A_1_, which is received by the transducer without entering the yeast suspension (i.e., sample). The transmitted portion of A_0_ propagates through the yeast suspension and is reflected at the sample–reflector interface, generating the second echo, E_2_, with amplitude A_2_. This echo traversed the sample twice (i.e., a propagation path within the sample of 2*d*) and carries information about the acoustic attenuation and transmission characteristics of the yeast suspension. A third echo, E_3_, with amplitude A_3_ results from an additional reflection cycle within the sample. Due to the extended propagation path within the sample of 4*d*, A_3_ experiences greater attenuation and may provide information about multiple scattering effects in the sample medium.

### Sample preparation

Wort solutions were prepared with extract concentrations of 10, 12, and 14 wt%. To do this, a commercially available malt extract (Bavarian pilsner, Weyermann^®^ GmbH, Bamberg, Germany) was diluted with demineralized water to the target wort concentrations. The required amount of malt extract was calculated based on the analytically determined concentration of the Bavarian pilsner malt extract.

Three commercially available dry yeast strains, with unique physiological properties and commonly used in the food and beverage industry, were selected for this study. Top-fermenting *Saccharomyces cerevisiae* (code: S. cerevisiae; FERMIPAN^®^ RED, Casteggio Lieviti srl, Casteggio, Italy) and *Saccharomyces cerevisiae* var. *diastaticus* (code: WB-06; SafAle™ WB-06, Fermentis, Division of S.I. Lesaffre, Marcq-en-Baroeul, France) were examined. A bottom-fermenting yeast, *Saccharomyces pastorianus* (code: W 34/70; SafLager™ W 34/70, Fermentis, Division of S.I. Lesaffre, Marcq-en-Baroeul, France) was also investigated. WB-06 is commonly used for wheat beer fermentation (e.g., hefeweizen or Belgian witbier). In contrast, W 34/70, a natural hybrid of *S. cerevisiae* and *S. eubayanus*, is the most widely used lager yeast in the brewing industry. All sealed dry yeast samples were stored at 4 °C until use.

To maintain the original physiological state and morphology of the yeast, and to prevent yeast proliferation and CO_2_ production during rehydration, dry yeast was first suspended in sterile 25% Ringer solution (i.e., electrolyte solution) for 30 min at 20 °C. Following rehydration, the suspension was centrifuged at 3000 rpm for 10 min at 20 °C. The supernatant was discarded, and the yeast was washed with the corresponding wort and centrifuged again. After washing, concentration gradients were prepared for each yeast by suspending the washed yeast in wort. A concentration gradient from 0.0 wt% to 1.0 wt% was prepared with 0.2 wt% increments, resulting in six concentration levels per strain. These gradients were prepared for each wort concentration (10, 12, and 14 wt% extract); each yeast suspension in the gradient was 250 mL in volume. For all sample increments, yeast cell count was determined using a hemocytometer with a chamber depth of 0.1 mm (Blaubrand, Sigma-Aldrich, St. Louis, MO, USA), following the procedures outlined in MEBAK sections 10.4.3.1 and 10.11.4.4 [38]. All yeast suspensions used in this study were produced in three biological replicates.

### Ultrasonic measurements

All samples across the concentration gradient (0.0–1.0 wt%), for each yeast strain at each wort concentration (10, 12, and 14 wt% extract), were measured in the experimental ultrasound setup at 20 °C. At each yeast concentration increment, 50 ultrasound signals were recorded in 0.5 s intervals, resulting in a total acquisition time of 25.0 s. Processing and feature extraction of the recorded raw ultrasound data, ${x}_{0}(t)$, were carried out as described below.

### Ultrasound data processing

Ultrasound data processing was performed using MATLAB R2023a (MathWorks, Natick, MA, USA).

#### Preprocessing of ultrasound signals

**Fig. 2:**
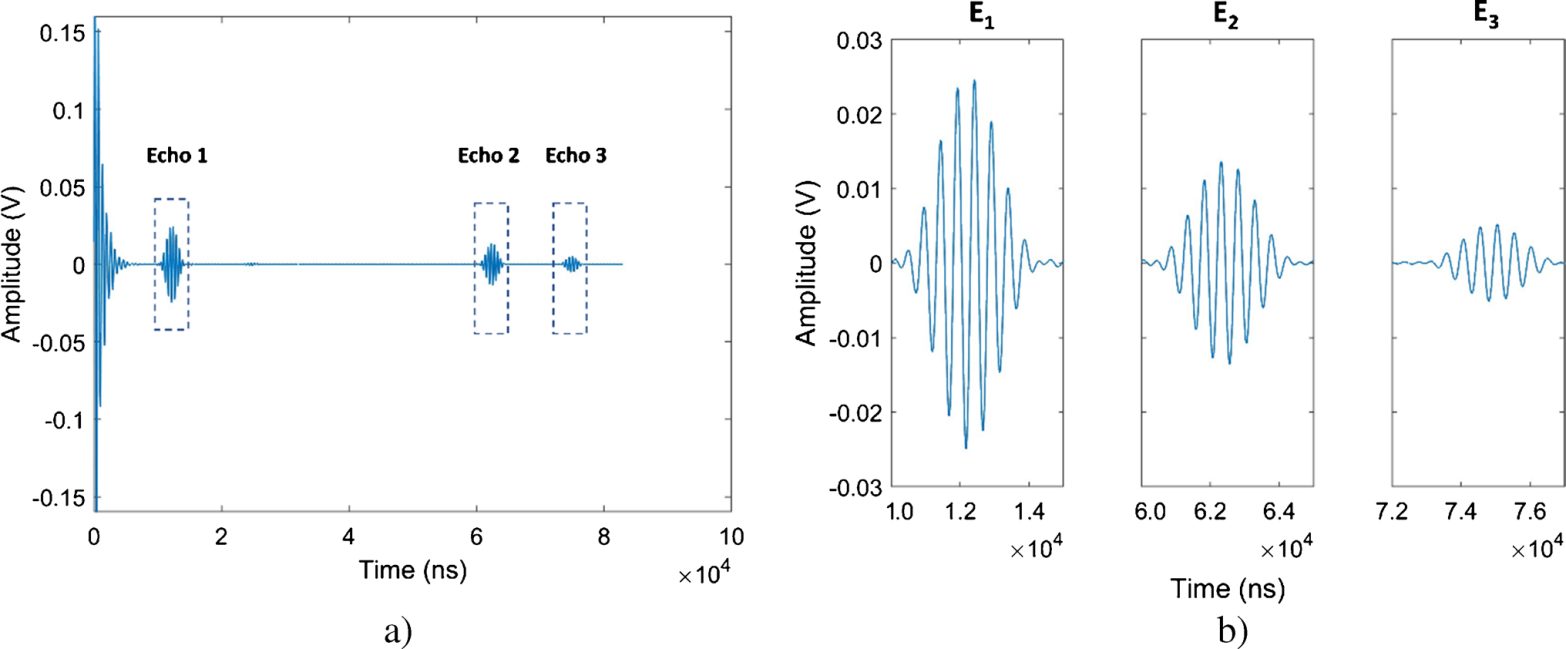
Representative ultrasound signal, $x\left(\text{t}\right)$, showing three detected echoes (E_1_, E_2_, and E_3_). The complete signal is shown in **a**, with the echo locations indicated by boxes. Enlarged views of each echo are included in **b**. The echoes were identified within predefined time intervals

The recorded raw data, ${x}_{0}(t)$, from the ultrasonic measurements were one-dimensional time series sampled at 1 GHz. To isolate the frequency range most responsive to the transducer, the signals were filtered using an eighth-order bandpass filter centered around 2 MHz (i.e., 1.5–2.5 MHz). Filtering was performed in a zero-phase manner to preserve the temporal structure of the signals and ensure sufficient attenuation of frequency components outside the passband [39]. The resulting filtered time domain signal retained the original echo structure. To enhance the signal-to-noise ratio, every five consecutive signals were averaged. This procedure improved signal consistency and suppressed random fluctuations, resulting in the ultrasound signal, $x\left(t\right)$; see Fig. 2.

#### Ultrasound feature extraction

To identify relevant echo characteristics, the envelope, $e\left(t\right)$, of each ultrasound signal, $x\left(t\right)$, was calculated. The envelope, $e\left(t\right)$, approximates the instantaneous amplitude of the signal and allows robust identification of reflected signal structures [40]. It was estimated by detecting the local maxima of the absolute signal within a moving window of 1000 sample points, which corresponds to 1000 ns at a 1-GHz sampling rate. This method effectively extracts the upper contour of the waveform and accentuates energy-rich regions, such as echoes. Three predefined time intervals, corresponding to the expected echo arrivals, were selected based on prior knowledge of the experimental setup geometry and signal propagation times; see Fig. 2b. The predefined time intervals selected for echo signal characterization were as follows:

- First echo, E_1_: [a_1_, b_1_] = [10,000, 15,000] ns
- Second echo, E_2_: [a_2_, b_2_] = [60,000, 65,000] ns
- Third echo, E_3_: [a_3_, b_3_] = [72,000, 77,000] ns

For each echo *i* ∈ {1, 2, 3}, the following three temporal and amplitude-related ultrasound signal features were extracted:

The maximum amplitude, ${A}_{max,i}$, of the envelope, $e\left(t\right)$, within the time interval [${a}_{i}$, ${b}_{i}$]:

1 $${A}_{max,i}=\underset{t \in \left[{a}_{i}, {b}_{i}\right]}{\text{max}}e\left(t\right).$$

The time, ${t}_{max,i}$, at which $e\left(t\right)$ reaches its maximum within the same interval:

2 $${t}_{max,i}=\underset{ t \in \left[{a}_{i}, {b}_{i}\right]}{\text{arg max}}\,\, e\left(t\right).$$

The sum, ${A}_{sum, i}$, of the absolute values of the signal $x\left(t\right)$ over the interval, which serves as a measure of signal intensity:

3 $${A}_{sum, i}=\sum_{t={a}_{i}}^{{b}_{i}}\left|x\left(t\right)\right|.$$

These ultrasound-derived features provide quantitative and qualitative information about the interaction of ultrasonic waves with the sequentially layered media of the experimental setup. The three recorded echoes differ in their propagation paths and physical origins. Therefore, the derived features (${A}_{max,i}$, ${t}_{max,i}$, and ${A}_{sum, i}$) capture specific acoustic interactions that depend on the media properties and the corresponding propagation path length. The extracted features of the first echo, E_1_, mainly represent the acoustic impedance contrast at the buffer rod–sample interface. In contrast, E_2_ and E_3_ traverse the yeast suspension and therefore carry information related to the attenuation and scattering properties of the suspension*.* The maximum amplitudes of E_2_ and E_3_ (${A}_{max,2}$ and ${A}_{max,3}$) reflect the local peak signal intensities, with higher yeast concentrations causing stronger attenuation and, consequently, lower amplitudes. Accordingly, the temporal positions of the echo maxima (${t}_{max,2}$ and ${t}_{max,3}$) are associated with the effective speed of sound and acoustic path length within the sample. The sums of the absolute values of E_2_ and E_3_ (${A}_{sum,2}$ and ${A}_{sum, 3}$) represent the total reflected acoustic energy, thereby integrating the effects of both attenuation and scattering-related signal broadening. Collectively, these features translate the raw ultrasound signals into quantitative descriptors of the examined yeast suspensions. This procedure yielded three features per analyzed echo, resulting in a total of nine ultrasound-derived features that were used as input features for subsequent model training and development.

### Artificial neural networks

**Fig. 3:**
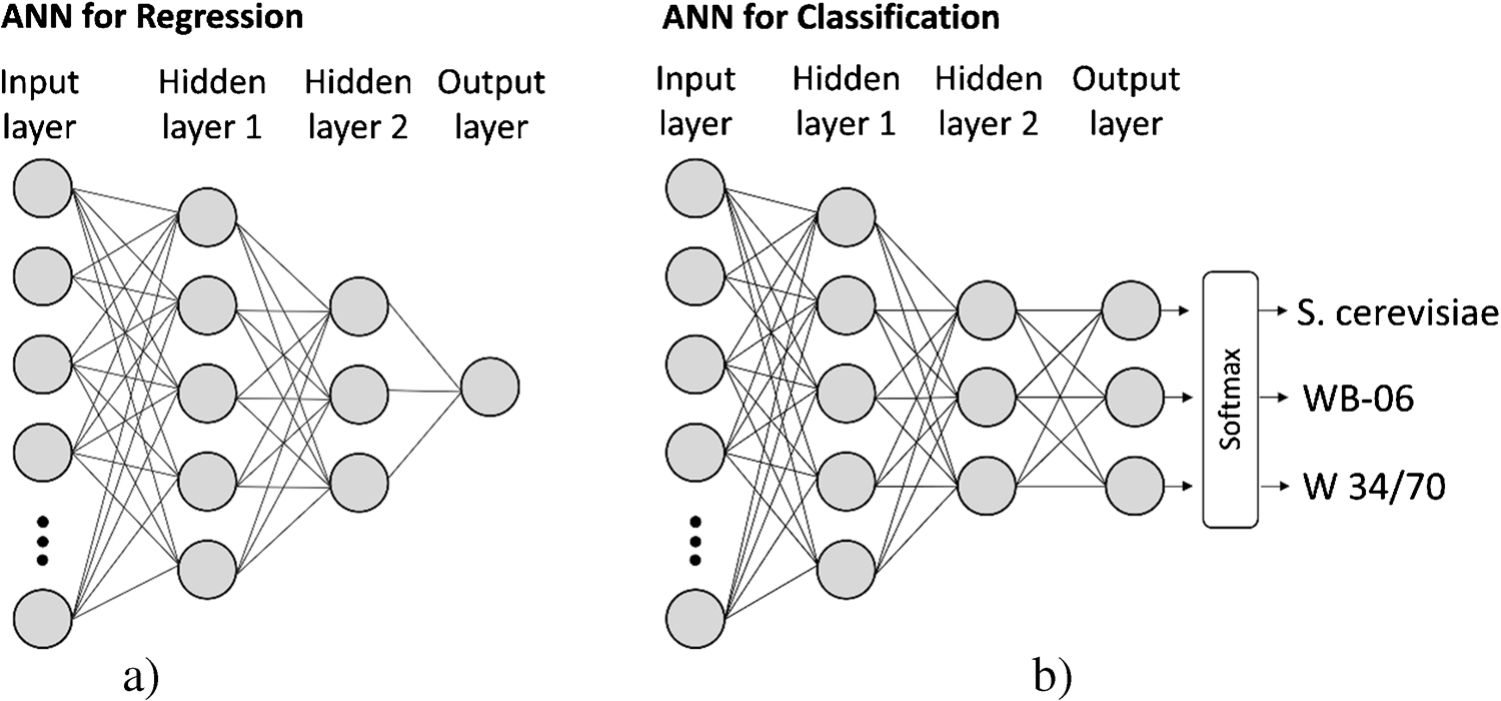
Comparison of the used basic artificial neural network architectures for regression (**a**) and classification (**b**). Both models have an input layer with 9–13 neurons, depending on the number of input features used in each approach (neurons partially shown; represented by gray circles), and two fully connected hidden layers with five and three neurons, respectively. The regression ANNs have a single output neuron suitable for predicting yeast cell count. The classification ANN includes three output neurons with a softmax activation function in the output layer, enabling multi-class classification of the yeast strains

Five artificial neural network modeling approaches were developed using MATLAB R2023a (MathWorks, Natick, MA, USA), and these are summarized in Tables 1 and 2. All ANN models were trained on a system equipped with an AMD Ryzen 7 5825U processor (2.0 GHz, 8 cores, 16 GB RAM) without GPU acceleration. Approaches 1–4 focused on predicting yeast cell counts using regression models (Fig. 3a), while Approach 5 was developed for the classification of samples by yeast strain (Fig. 3b). The regression models differed in the manner in which nominal categorical (strain identity) and ordinal (wort concentration) information were incorporated as model inputs. Strain identity was excluded entirely in Approaches 1 and 2 but included as an encoded input feature in Approaches 3 and 4. In Approach 5, by contrast, strain identity was used as the prediction target in a multi-class classification model. However, all ANNs shared the same basic network architecture with two hidden layers and used a data split of 80:20 for training and testing. Model performance was assessed using fivefold cross-validation on the training set and subsequently evaluated independently on the test set.

**Table 1:** Overview of artificial neural network (ANN) hyperparameters and training settings

Parameter	Settings	Description
Number of hidden layers	2	Compact architecture chosen to balance model complexity and generalization
Neurons per layer	5 (layer 1); 3 (layer 2)	Selected based on cross-validation performance and model stability
Activation function	ReLU	Applied to both hidden layers
Regularization (L2, λ)	0.001	Used to limit overfitting and improve generalization
Optimizer	L-BFGS	Deterministic full-batch optimization and fast convergence
Batch size	Full batch	Used for deterministic gradient updates
Early stopping patience	6 epochs	Training terminated when validation loss plateaued
Loss function	MSE (regression); cross-entropy (classification)	Selected according to the respective modeling objective
Cross-validation	Fivefold	Used to estimate model variance and evaluate generalization performance

A compact, fully connected artificial neural network with two hidden layers was applied to both regression and classification models, differing in the loss function and output layer. The reported settings were selected based on preliminary modeling to ensure a robust balance between predictive accuracy and generalization performance

**Table 2:** Overview of the artificial neural network (ANN) approaches used

	Task type	Dataset	Input features	Prediction target	Encoding	Description
Approach 1	Regression	Three strain-specific datasets	9 ultrasound features	Cell count (continuous)	–	Strain-specific models trained separately for each yeast strain
Approach 2	Regression	Combined dataset (all strains)	9 ultrasound features	Cell count (continuous)	–	General regression model trained across all strains without categorical or process-related inputs
Approach 3	Regression	Combined dataset (all strains)	9 ultrasound features + 3 strain identity indicators	Cell count (continuous)	One-hot encoding (strain identity)	Model including nominal strain identity to enhance generalization
Approach 4	Regression	Combined dataset (all strains)	9 ultrasound features + 3 strain identity indicators + 1 wort concentration variable	Cell count (continuous)	One-hot encoding (strain identity); ordinal encoding (wort concentration)	Extension of Approach 3, including the ordinally encoded wort concentration
Approach 5	Classification (softmax output)	Combined dataset (all strains)	9 ultrasound features	Yeast strain (categorical)	–	Classification model distinguishing between yeast strains

Approaches differ in task type, dataset, input feature composition, prediction target, and encoding strategy. The nine ultrasound-derived input features were used in all models. Strain identity, a nominal categorical variable, was encoded using one-hot encoding (S. cerevisiae* as* [1 0 0]; WB-06 as [0 1 0]; and W 34/70 as [0 0 1]). Wort concentration (10, 12, and 14 wt%) was encoded as a single ordinal variable with numeric values of −1, 0, and +1, respectively. Regression models predicted continuous yeast cell counts, while the classification model distinguished between yeast strains

#### Model architecture and data management

Preliminary modeling included systematic testing of different network depths (1–3 hidden layers), numbers of neurons per layer (5–30), and L2 regularization strengths (*λ* = 0.0–0.1). Increasing model complexity reduced training bias but led to a widening discrepancy between training and test errors, indicating a tendency toward overfitting. Therefore, a compact, fully connected artificial neural network with two hidden layers (five and three neurons) was selected as the basic network architecture to balance model complexity and generalization performance. The rectified linear unit (ReLU) activation function was applied to all hidden layers. In addition, L2 regularization (*λ* = 0.001) was applied to all models to prevent overfitting by penalizing large network weights. This regularization term helped stabilize training and improve generalization performance. Prior to training, all ultrasound-derived input features were standardized to zero mean and unit variance. The regression models minimized mean squared error (MSE), while the classification model was trained using cross-entropy loss. Model optimization employed the limited-memory Broyden–Fletcher–Goldfarb–Shanno (L-BFGS) algorithm, and network weights were initialized with Glorot uniform initialization [41]. To prevent overtraining, early stopping with a validation patience of six epochs was implemented, halting training when performance no longer improved. The network architecture, hyperparameters, and training settings used are summarized in Table 1.

For each strain, the dataset used for regression model training included the manually determined cell counts and nine ultrasound-derived features from all individual samples in the yeast suspension concentration gradients, with each sample assessed at three different wort concentrations. These three strain-specific datasets (used in Approach 1) were then merged to form the combined dataset (used in Approaches 2–5). Therefore, the combined dataset consisted of three identically structured sets, each corresponding to a different yeast strain. Approaches 1–4 were developed as regression models for yeast cell count prediction, with Approach 1 trained on strain-specific datasets and Approaches 2–4 trained on the combined dataset. While Approach 2 used only ultrasound-derived features as inputs, Approaches 3 and 4 progressively incorporated additional encoded variables, beginning with nominal strain identity and followed by ordinal wort concentration. In contrast, Approach 5 was developed as a classification model to distinguish between yeast strains. An overview of all approaches is provided in Table 2.

**Fig. 4:**
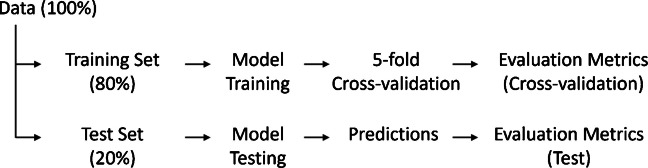
Overview of data partitioning and evaluation procedure. The data (100%) were split into a training set (80%) and a test set (20%). The training set was used for model training with fivefold cross-validation to assess model performance. Final model performance was evaluated on the independent test set

Each dataset (strain-specific or combined), with its respective input features and prediction target, was randomly partitioned into 80% for training and 20% for independent testing (see Fig. 4). Within the training set, fivefold cross-validation was applied to evaluate model performance and generalization. Test data were not used during training, thereby ensuring unbiased model performance assessment. The evaluation metrics used are described in the section “Model Evaluation Metrics.”

#### Regression models for yeast cell count prediction (Approaches 1–4)

##### Approach 1: Strain-specific regression

An independent model was trained for each yeast strain, resulting in three separate models for yeast cell count prediction. Approach 1 represents a use case where the strain identity (e.g., W 34/70) is known at prediction time. Therefore, each model was trained independently using only the corresponding yeast strain training set (80% of the data), with the nine ultrasound-derived features as input, each corresponding to one neuron in the input layer. Approach 1 handled three smaller datasets and allowed models to specialize in strain-specific patterns for cell count prediction.

##### Approach 2: General regression model

In Approach 2, the combined dataset, formed by merging three strain-specific yet identically structured datasets, was used to develop a single regression model able to predict yeast cell count for all strains. This ANN used only the nine input features derived from ultrasound echoes, with the manually determined yeast cell counts as the prediction target. This general modeling approach provided an initial assessment of the predictive power of the ultrasound-derived features alone for yeast cell count prediction. Additional input features, such as strain identity and wort concentration, which represent biological differences and process variability, respectively, were not included.

##### Approach 3: Regression with one-hot encoding of strain identity

Approach 3 expands on Approach 2 by introducing strain identity as a nominal categorical variable via one-hot encoding [42]. Three binary indicators were used to encode the strain identity: S. cerevisiae as [1 0 0], WB-06 as [0 1 0], and W 34/70 as [0 0 1]. These one-hot encoded vectors added three input neurons to the model and were concatenated with the nine original ultrasound-derived features. Therefore, the regression model of Approach 3 used a total of 12 input neurons. One-hot encoding was chosen since the nominal nature of the strain identity indicates that the classes have no inherent order or hierarchy. Therefore, Approach 3 allowed the model to predict yeast cell counts across all strains while accounting for strain-specific differences.

##### Approach 4: Regression with one-hot encoding of strain identity and ordinal encoding of wort concentrations

This modeling approach for yeast cell count prediction incorporated two additional non-ultrasound input features: strain identity and wort concentration. Strain identity was treated as a nominal categorical variable and encoded using one-hot vectors ([1 0 0], [0 1 0], [0 0 1]), while wort concentrations of 10, 12, and 14 wt% were encoded as an ordinal variable with values of −1, 0, and +1, respectively. Unlike the nominal nature of strain identity, the ordinal encoding of wort concentration reflects an inherent order. This allowed the model to account for both nominal categorical variation (strain identity) and ordered process variability (wort concentration), thereby improving its ability to learn from the underlying relationships in the data. Therefore, a total of 13 input features were used: nine ultrasound-derived features, three binary indicators for strain identity, and one numeric variable for wort concentration. Each input feature corresponded to one neuron in the input layer. Among the regression models, this configuration provided the highest input complexity and specificity and was expected to improve generalization across different strains and wort concentrations.

#### Yeast strain classification model (Approach 5)

##### Approach 5: Classification of yeast strains

In this approach, a classification model was trained to predict strain identity (i.e., class membership) based solely on the nine ultrasound-derived features. This model shared the same basic network architecture as Approaches 1–4, but differed in the output layer, which employed a softmax function for multi-class classification of yeast strains (see Fig. 3b). This approach served as a verification model, designed to assess whether the ultrasound-derived features align with the expected strain classification.

#### Model evaluation metrics

Model evaluation metrics were calculated using fivefold cross-validation on the training set and subsequently evaluated on the independent test set. Cross-validation provided a general estimate of model performance, while the test set allowed for independent assessment of generalization to unseen data. For the regression models (i.e., Approaches 1–4), model performance was assessed using the coefficient of determination (*R*^2^), root mean square error (RMSE), and mean absolute error (MAE):

4 $${R}^{2}=1- \frac{\sum_{i=1}^{n}{\left({y}_{i}-{\widehat{\text{y}}}_{i}\right)}^{2}}{\sum_{i=1}^{n}{\left({y}_{i}-{\overline{\text{y}} }\right)}^{2}},$$

5 $$RMSE=\sqrt{\frac{1}{n} \sum_{i=1}^{n}{\left({y}_{i}-{\widehat{\text{y}}}_{i}\right)}^{2}},$$

6 $$MAE=\frac{1}{n}\sum_{i=1}^{n}\left|{y}_{i}-{\widehat{\text{y}}}_{i}\right|,$$

where ${y}_{i}$ is the measured (i.e., true) value, ${\widehat{\text{y}}}_{i}$ is the predicted value, ${\overline{\text{y}}}$ is the mean of the measured values, and $n$ is the number of data points.

For the classification approach (i.e., Approach 5), the model performance was assessed using overall accuracy, defined as the percentage of correct predictions. Additionally, model performance metrics for each yeast strain class, including precision, recall, and F1-score, were calculated as follows:

7 $$Precision=\frac{TP}{TP+FP},$$

8 $$Recall=\frac{TP}{TP+FN},$$

9 $$\mathit{F1\text{-}score} = 2 \cdot \frac{\mathit{Precision} \cdot \mathit{Recall}}{\mathit{Precision} + \mathit{Recall}}$$

True positives (TP) indicate the number of instances correctly classified as belonging to their actual yeast strain class, and false positives (FP) represent the number of instances incorrectly classified as belonging to a particular strain when these belong to another. False negatives (FN) are instances from a given yeast strain that were misclassified as belonging to a different strain. These metrics evaluate the performance of the classification model in distinguishing between yeast strains.

## Results and discussion

### Regression models for yeast cell count prediction

Three different yeast strains and three wort concentrations were selected to produce concentration gradients with varying yeast cell counts. As a result, each sample had a unique composition, potentially affecting ultrasonic wave propagation in distinct patterns. To introduce biological variation, S.cerevisiae, WB-06, and W 34/0 were chosen, whereas increasing wort concentrations of 10, 12, and 14 wt% were used to represent variability in process conditions common in fermentation processes. An experimental ultrasound setup was used for noninvasive acquisition of ultrasound signals propagating through the samples. Subsequently, nine ultrasound-derived features were used as input features to train and develop artificial neural network models for yeast cell count prediction. To provide an unbiased evaluation of the models, the predictive performance of the regression models (Approaches 1–4) was assessed using the unseen test set. All parity plots shown correspond to the test set predictions. In Approaches 1–4, fully connected neural networks were trained to identify ultrasound-based patterns and to learn relevant relationships for reliably predicting yeast cell count.

#### Strain-specific regression (Approach 1)

**Fig. 5:**
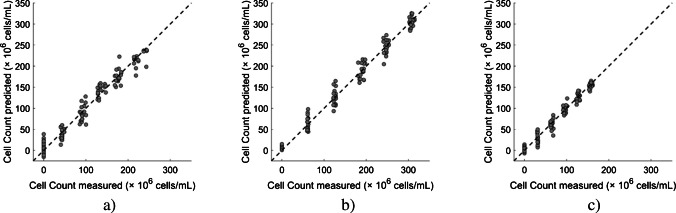
Parity plots (test set) comparing measured and predicted yeast cell count using ANN regression (Approach 1) for three yeast strains: S. cerevisiae (**a**), WB-06 (**b**), and W 34/70 (**c**). The dashed diagonal line represents the ideal parity where predicted values equal measured values. Closer alignment of data points to the diagonal indicates better predictive performance. Artificial neural network strategies are described in Table 2

In Approach 1, all three models were developed using the same ANN configuration but were trained and evaluated separately on strain-specific data. The performance of these strain-specific models is shown in Fig. 5 using three parity plots, each representing one of the examined yeast strains. Each parity plot compares the predicted and manually measured yeast cell counts. The closer the data points are to the ideal parity (i.e., predicted = measured), represented by the dashed diagonal line, the better the model performance. The corresponding histograms of prediction errors also indicate the adequacy of the regression model fit (see Electronic Supplementary Material, Fig. S1). In addition, for Approaches 1–4, the regression model evaluation metrics, including the coefficient of determination, root mean square error, and mean absolute error, for both cross-validation and independent test sets are summarized in Table 3. Higher *R*^2^ values and lower RMSE and MAE values indicate better predictive performance.

**Table 3:** Model evaluation metrics

	Cross-validation			Test		
Model	*R*^2^	RMSE (10^6^ cells/mL)	MAE (10^6^ cells/mL)	*R*^2^	RMSE (10^6^ cells/mL)	MAE (10^6^ cells/mL)
Approach 1 S. cerevisiae	0.96	16.3	12.3	0.96	15.9	12.6
Approach 1 WB-06	0.98	14.3	10.7	0.98	14.6	11.3
Approach 1 W 34/70	0.97	9.0	6.7	0.97	9.5	7.4
Approach 2	0.94	22.2	16.7	0.92	24.0	18.1
Approach 3	0.97	15.0	11.3	0.96	16.6	12.3
Approach 4	0.98	13.2	9.7	0.97	14.8	10.7

Cross-validation and test performance metrics for all ANN regression models (Approaches 1–4). Metrics reported are the coefficient of determination (*R*^2^), root mean square error (RMSE), and mean absolute error (MAE). Artificial neural network strategies are described in Table 2

For all Approach 1 models, high predictive accuracy was evident from the dense data clustering and was confirmed by the model performance metrics (see Table 3). Although all Approach 1 regression models performed well, W 34/70 achieved an *R*^2^ of 0.97 and yielded the smallest test error margins (RMSE = 9.5 × 10^6^ cells/mL; MAE = 7.4 × 10^6^ cells/mL). Compared with W 34/70, which did not exceed 165 × 10^6^ cells/mL, higher cell counts were recorded for S. cerevisiae and WB-06, reaching maximum concentrations of approximately 245 × 10^6^ cells/mL and 315 × 10^6^ cells/mL, respectively. Despite these strain-specific differences, these results confirm that the used basic neural network architecture performed well across different yeast strains and cell count ranges. Moreover, the variation in RMSE and MAE could indicate differences in acoustic scattering properties influenced by strain-dependent factors, like cell size and morphology. Nonetheless, the consistently high *R*^2^ values for all yeast strains suggest that the predictive models effectively captured the underlying relationships between ultrasound-derived features and yeast cell count.

#### Progressive improvement of regression models for yeast cell count prediction (Approaches 2–4)

**Fig. 6:**
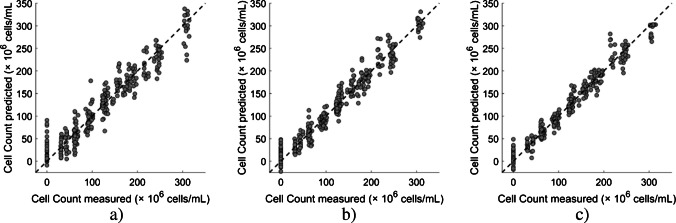
Parity plots (test set) of measured versus predicted yeast cell count for three ANN regression approaches: Approach 2 (**a**), Approach 3 (**b**), and Approach 4 (**c**). The dashed diagonal line represents the ideal parity where predicted values equal measured values. Closer alignment of data points to the diagonal indicates better predictive performance. Artificial neural network strategies are described in Table 2

Unlike Approach 1, in which three strain-specific models were developed, Approaches 2–4 were trained using the combined dataset, which comprised the three identically structured, strain-specific sets. These models shared the same basic ANN architecture; however, each subsequent model progressively incorporated additional non-ultrasound input features of increasing specificity, resulting in one single model per approach. To visualize the model performance of Approaches 2–4 for yeast cell count prediction, the parity plots are presented in Fig. 6, and the corresponding histograms of prediction errors are provided in the Electronic Supplementary Material (Fig. S2).

Although all models shared the same underlying network architecture, the input features in Approaches 2–4 differed in number and specificity, ranging from low (nine input features) to high (13 input features), as well as from general (Approach 2) to detailed (Approach 4). As expected, Approach 2 exhibited the highest deviation from the parity line, indicating lower predictive performance, particularly at higher yeast cell counts (300–315 × 10^6^ cells/mL). Conversely, as the input information became increasingly structured and encoded, a progressive improvement in the prediction performance of Approaches 3 and 4 was observed and confirmed by the model evaluation metrics (see Table 3). In Approach 3, the cell count prediction accuracy increased with the inclusion of strain identity as a nominally encoded input, but the best performance was obtained with Approach 4 by additionally incorporating wort concentration as an ordinally encoded input feature. Unlike the parity plot of Approach 2, the yeast cell count predictions from the other two regression models (Approaches 3 and 4) showed a closer alignment with the measured values across all concentrations. When using a regression model for all yeast strains, the most robust and reliable yeast cell count predictions were achieved with Approach 4, as confirmed by the parity plots and the test performance metrics (*R*^2^ = 0.97, RMSE = 14.8 × 10^6^ cells/mL, and MAE 10.7 × 10^6^ cells/mL). These results indicate that although all predictive models captured the underlying relationship between the input features and cell count, both accuracy and performance improved progressively from Approach 2 to Approach 4. This further emphasizes the importance of appropriate input feature selection, robust training procedures, and neural network architecture in regression modeling. Similar performance trends were observed when comparing the model evaluation metrics from the cross-validation to those of the test sets (see Table 3). The comparable or marginally better cross-validation performance suggests that the developed regression models did not overfit. Moreover, the ability of these models to generalize well to unseen data further supports the robustness of both the training procedures and the selected ANN architectures.

### Yeast strain classification model (Approach 5)

**Fig. 7:**
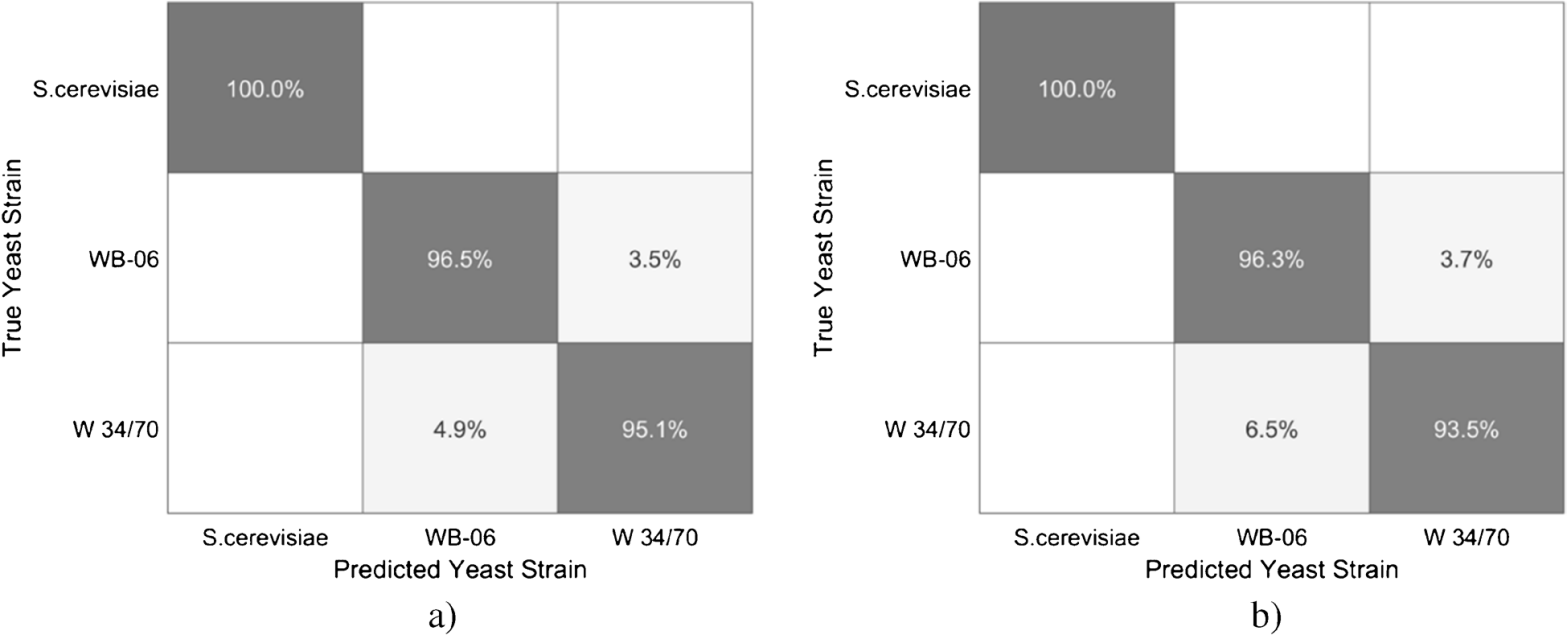
Confusion matrices showing the performance of yeast strain classification (Approach 5) for cross-validation (**a**) and the test set (**b**). Predicted classes are shown on the horizontal axis, and true classes on the vertical axis. Values represent the percentage of samples classified into each yeast strain

A separate classification model was developed to distinguish between the three yeast strains (i.e., S. cerevisiae, WB-06, or W 34/70) based solely on the nine ultrasound-derived input features. This classification model shared the same basic architecture as Approaches 1–4 but included a softmax activation function in the output layer, enabling multi-class yeast strain classification. Figure 7 shows the confusion matrices for both cross-validation (Fig. 7a) and the test set (Fig. 7b). The corresponding model performance metrics, including precision, recall, and F1-score, are summarized in Table 4.

**Table 4:** Model evaluation metrics

	Cross-validation			Test		
	Precision (%)	Recall (%)	F1-score (%)	Precision (%)	Recall (%)	F1-score (%)
S. cerevisiae	100.0	100.0	100.0	100.0	100.0	100.0
WB-06	95.2	96.5	95.9	93.7	96.3	94.9
W 34/70	96.5	95.1	95.8	96.2	93.5	94.8

Cross-validation and test performance metrics for yeast strain classification (Approach 5). Metrics reported are precision, recall, and F1-score. Artificial neural network strategies are described in Table 2

The model developed in Approach 5 achieved an overall classification accuracy of 97.2% during cross-validation and 96.6% on the independent test set, thereby confirming its strong generalization performance. The dark gray diagonal cells in the confusion matrices represent correct classifications, while the other cells indicate misclassifications.S. cerevisiae was correctly classified in all cases (100%) across both the cross-validation and test evaluations. For WB-06 and W 34/70, only a small percentage of samples were incorrectly classified, 3.5–3.7% for WB-06 and 4.9–6.5% for W 34/70. These results confirm that Approach 5 accurately classified yeast strains based solely on ultrasound-derived features. Moreover, the consistently high F1-scores across all yeast strains emphasize the robustness of this classification model and its suitability for reliable yeast strain identification. Similar to the regression models (Approaches 1–4) used for yeast cell count prediction, only minor differences were observed in the evaluation metrics of the classification model (Approach 5) between cross-validation and the independent test set.

### Regression and classification approaches for bioprocess monitoring

In this study, all regression and classification models were developed and trained using widely accessible computational resources, with each model requiring less than 5 s to complete training. Model performance metrics confirmed that the regression models successfully predicted yeast cell counts up to 315 × 10^6^ cells/mL, while the classification model reliably distinguished between the three yeast strains with an overall accuracy of 96.6%. However, their generalization beyond the current benchtop setup and experimental parameters should be examined. Therefore, to represent a wider range of fermentation conditions, the number of samples in the yeast concentration gradient should be increased (e.g., using 0.1 wt% increments) and tested at higher and lower wort concentrations. Furthermore, determining their adaptability and consistent performance across different applications requires systematic testing under varied biological and process conditions. Consequently, future studies should consider a broader selection of yeast strains, a wider range of substrate compositions (e.g., different wort concentrations), and multiple process phases to comprehensively evaluate model robustness and transferability.

While the physical properties of the medium are directly affected by temperature, an additional challenge arises from the dynamic nature of yeast suspensions during fermentation. Compositional variations caused by common metabolic products, such as carbon dioxide and ethanol, alter the density and compressibility of the medium. All these factors directly influence ultrasound propagation behavior and signal quality, and therefore must be carefully accounted for. To further improve the prediction and generalization performance of the models, these signal-affecting variables or an outlier detection function could be incorporated into the basic artificial neural network [43]. Alternatively, incorporating additional ultrasound-derived features, such as amplitude or energy ratios between echoes, may enhance sensitivity to subtle changes in yeast cell properties or medium composition.

Although the ANN regression models achieved low bias and high predictive accuracy, some variance remained across samples. In the present study, variance was controlled through fivefold cross-validation, L2 regularization, and early stopping. These methods were effective in ensuring stable model performance while preventing overfitting. To improve future model development, alternative variance reduction strategies, such as dropout regularization, ensemble learning, or noise injection during training, should be investigated [44]. Such strategies could further enhance model robustness and generalization, particularly when applied to larger and more heterogeneous datasets.

Additionally, the analytical dataset could be refined. Dimensionality reduction techniques like principal component analysis or autoencoders could be used to reduce the feature space and the risk of overfitting, while also improving interpretability [37]. Furthermore, the application of explainable artificial intelligence (XAI) tools can help identify the most important input features [45, 46]. This, in turn, can provide additional process insights and enhance process understanding, both considered important prerequisites for industrial applications. In future work, sophisticated interpretability methods, such as SHAP (SHapley Additive exPlanations) [47, 48] and permutation importance [49], could be employed to assess the contribution of individual ultrasound-derived and encoded features to model predictions. Complementary analyses, including partial dependence or accumulated local effects (ALE) plots, could further reveal how variations in specific features affect model behavior [48, 50]. Feature ablation or gradient-based sensitivity analyses [50, 51] may further support these findings by relating data-driven interpretations to the underlying physical phenomena of ultrasound propagation, such as attenuation and scattering.

The present study focused on benchtop experiments; however, the proposed approach demonstrates considerable potential for implementation in large-scale operational environments. For practical online implementation, ultrasound sensors need to be integrated into pipelines or fermenters to allow continuous, noninvasive monitoring. The use of a Varivent^®^ process connector in this experimental setup already addresses industrial design principles, as it allows hygienic integration of measurement and control instruments. However, integration into operational environments still poses several challenges. Sensor fouling is likely to occur in wort- or yeast-rich media [52], which may reduce ultrasound signal quality over time and require reliable cleaning strategies [53] or self-diagnostic functions [54]. Furthermore, establishing online measurements requires efficient signal processing pipelines and seamless connectivity with existing process control architectures, such as SCADA (supervisory control and data acquisition) systems or industrial Internet of Things (IoT) platforms [55]. Addressing these challenges is essential for developing robust online systems that allow industrial process monitoring and control. Online monitoring of yeast cell count and strain identity could support real-time control of fermentation kinetics and detection of contaminations, thereby improving process reliability and optimizing product yield. Moreover, integrating ultrasound-based monitoring approaches into process control systems would facilitate data-driven automation and contribute to enhanced product quality and process efficiency.

## Conclusion and outlook

Continuous monitoring of yeast cell count contributes to reproducible and consistent quality by maximizing process efficiency and minimizing variations. Established methods are typically either off-line or, when applied in-line, often invasive. These approaches have specific limitations for continuous industrial operations, such as delayed feedback and potential contamination risks. Conversely, ultrasound methods are noninvasive and allow real-time monitoring. In this study, a representative dataset, which covers variations in biological properties, process parameters, and their interactive effects on ultrasonic wave propagation, was investigated. Manually determined cell counts and nine ultrasound-derived features were used to train and develop separate ANN models for yeast cell count prediction.

In Approach 1, individual models were developed for each *Saccharomyces* strain; these revealed strain-specific patterns and confirmed that the selected ANN architecture performs well in predicting yeast cell count. Subsequently, the strain-specific data were merged to develop a general model able to predict yeast cell count across all strains. In Approaches 2–4, different ANN strategies were tested to progressively improve the predictive performance of the developed regression models. The model evaluation metrics revealed that accuracy and performance improved with the integration of non-ultrasound data (e.g., encoded nominal or ordinal variables). These findings confirmed the importance of the ANN architecture and emphasized the influence of input feature complexity and specificity on regression modeling. In addition, a separate model (Approach 5) was trained for yeast strain classification using only the nine ultrasound-derived features.

Within the tested conditions and parameters at the benchtop scale in this study, ultrasonic measurements and the developed models successfully predicted yeast cell counts up to 315 × 10^6^ cells/mL and classified the three yeast strains with high accuracy (>96%). This study provides insights into how ultrasound-based, ANN-driven modeling can deliver real-time feedback, thereby enabling advanced bioprocess monitoring. Future research focusing on the continued expansion of the underlying dataset, refinement of modeling strategies, and testing under industrial process conditions will further support integration into automated control systems.

## Supplementary Information

Below is the link to the electronic supplementary material.

Supplementary file1 (DOCX 207 KB)

## Data Availability

The data that support the findings of this study are available from the corresponding author upon request.
