# Supplementary material for: Noninvasive monitoring and prediction of cell count of Saccharomyces suspensions using ultrasonic measurements and artificial neural networks
Source: Anal Bioanal Chem. 2025 Nov 21;417(30):6875–89. doi: 10.1007/s00216-025-06175-6 (PMC12680890; doi:10.1007/s00216-025-06175-6)
Supplement: Supplementary file 1 — Supplementary file1 (DOCX 207 KB) [file 216_2025_6175_MOESM1_ESM.docx]

**Electronic Supplementary Material**


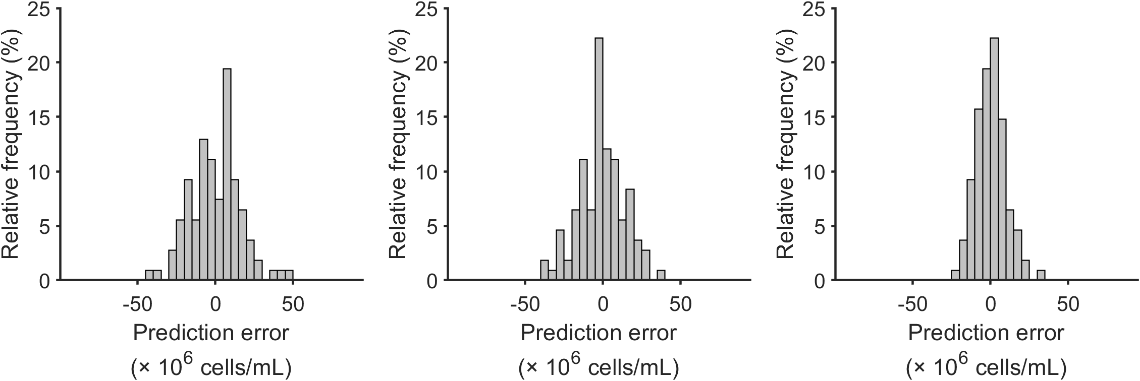


1. b) c)

Fig. S1 Histograms of prediction errors (test set) for the ANN regression models of Approach 1: S. cerevisiae (a), WB-06 (b), and W 34/70 (c). The residuals are symmetrically distributed around zero, exhibit approximate normality, and display no pronounced skewness or excess kurtosis, thereby supporting the adequacy of the regression model fit.


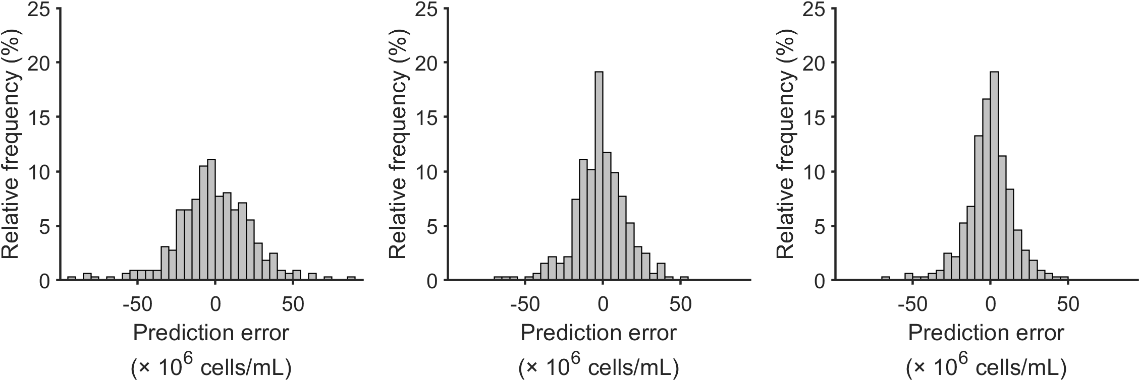


1. b) c)

Fig. S2 Histograms of prediction errors (test set) for three ANN regression approaches: Approach 2 (a), Approach 3 (b), and Approach 4 (c). The residuals are symmetrically distributed around zero, exhibit approximate normality, and display no pronounced skewness or excess kurtosis, thereby supporting the adequacy of the regression model fit.
